# Metagenomic insights into carbon and nitrogen cycling functions in an integrated rice-crayfish farming system with straw return

**DOI:** 10.3389/fmicb.2026.1940285

**Published:** 2026-10-26

**Authors:** Fen Wang, Zhu Chen, Xiang Zhou, Xuezhen Liu, Yangyang Jiang, Guoxia Fang, Youfeng Su, Chaozhen Rong, Suofei Ji, Guanjun Hou

**Affiliations:** 1 Key Laboratory of Aquaculture and Stock Enhancement for Anhui Province, Fisheries Research Institute, Anhui Academy of Agricultural Sciences, Hefei, China; 2 Feixi Longguanhe Ecological Agriculture Co., Ltd., Hefei, China; 3 Anhui Jie & Xiang Aquaculture Co. Ltd, Hefei, China; 4 Tongcheng Sushi Agricultural Development Co., Ltd., Tongcheng, China; 5 Hefei Animal Husbandry and Aquatic Products Technology Extension Center, Hefei, China

**Keywords:** C cycling, metagenomics, N cycling, rice-crayfish integrated system, straw return

## Abstract

Straw return is an important strategy for agricultural residue utilization, and functional microbial communities are known to play a pivotal role in straw decomposition and nutrient cycling. Nevertheless, the decomposition dynamics and associated microbial functional outcomes in rice-aquaculture systems remain poorly documented. This study established a rice-crayfish integrated system with straw return and assessed the mechanisms of straw decomposition and the resulting improvement on soil fertility through soil physicochemical characterization, enzyme activity assays, and metagenomic sequencing. Soil samples were obtained in July (PF7, the rice transplanting stage following the crayfish farming period) and November (PF11, the post-harvest field drying period), with surrounding ditches as controls (CK7 and CK11). The results showed that straw was effectively decomposed, releasing potassium and nitrogen nutrients, while urease and cellulase activities were markedly increased in the paddy field. Metagenomic analysis revealed that Pseudomonadota was the dominant phylum, whereas Euryarchaeota and Nitrososphaerota exhibited the most pronounced changes, with their abundances in PF11 being 0.23-fold and 10.57-fold of those in CK11, respectively. In the C cycle, C degradation was the most abundant pathway, in which amino acid degradation (primarily derived from crayfish farming) dominated, followed by aromatic compound degradation (associated with straw decomposition). For the methane metabolic pathway, the greenhouse gas emission potential was reduced, as evidenced by lower *mcrA* abundances in PF than in CK. N cycling was primarily mediated through organic N metabolism (81.63–84.14%), with the remaining fraction dominated by nitrification and denitrification. Furthermore, the relevant analysis consistently indicated that C cycling genes occupy more central positions and TN showed a negative effect on these genes. Collectively, this integrated system was associated with straw decomposition, facilitated terminal organic C and N mineralization, while greenhouse gas-related functional potentials were reduced, supporting sustainable straw utilization in rice-crayfish agroecosystems.

## 1 Introduction

China produces approximately 0.2 billion tons of rice straw annually (Liu L. et al., 2026), creating a large agricultural residue stream that entails both management challenges and resource-use opportunities. Direct straw incineration can suppress pests and return limited nutrients to soil in the form of ash. However, this practice removes most straw-derived nutrients and soil microorganisms and causes severe air pollution. Therefore, it has been banned by the Chinese government (Yang et al., 2026; Peng et al., 2024). Thus, the search for alternative straw management strategies has become a major research focus. Data from China’s Ministry of Agriculture and Rural Affairs (MARA) demonstrate that the comprehensive utilization rate of straw reached 88.1% in 2021, with 60% being used for fertilization, indicating that fertilizer-oriented use is the dominant straw utilization route in China (Zhao Y. et al., 2026). As straw is mainly composed of lignin, cellulose, and hemicellulose, insufficient decomposition may adversely affect crop health and yield stability (Gao et al., 2023). As a crop nutrient source, straw can reduce chemical fertilizer inputs and agricultural costs (Han et al., 2025). Nevertheless, direct straw return may create agronomic risks by introducing pathogens and pest eggs into soil (Liu L. et al., 2026). It may also increase greenhouse gas emission potential (Zhou et al., 2025).

To overcome the limitations of conventional straw return, recent studies have proposed multiple management strategies, including deep burial (Zhao D. et al., 2026), piled straw return (Liu Y. et al., 2026), and the application of decomposition agents, humic acid, and microbial consortia in paddy fields (Han et al., 2024; Peng et al., 2024; Pan et al., 2021). Among these approaches, the integration of straw return with organic fertilizer has attracted particular interest because of its potential to maintain soil multifunctionality and support sustainable agricultural ecosystems (Wu et al., 2026). Generally produced from livestock manure through aerobic composting, organic fertilizers supply active enzymes originating from animal digestion and microbially derived activity, and their application can rapidly increase soil enzyme diversity and abundance (Peng et al., 2024). In addition, straw return improves the availability of key nutrients and stimulates the proliferation and metabolic function of specific soil microbial communities. These microbial responses promote the secretion of enzymes involved in organic matter decomposition (Yan et al., 2026). When straw return is combined with organic fertilizer, the two inputs can have synergistic effects. By enhancing soil enzyme activity, straw and organic fertilizers jointly regulate carbon-nitrogen cycling, promote organic phosphorus mineralization and nitrogen transformation (Zhao et al., 2025a). During composting, actinomycetes, fungi, and bacteria secrete enzymes, including cellulases and lignin peroxidases, which accelerate lignocellulose degradation, promote humus formation, and reduce greenhouse gas emissions through microbial community modulation (Zhou et al., 2025; Zhao et al., 2025b).

Integrated rice-aquaculture farming has become a sustainable agricultural model actively promoted in China. In 2024, the national area under this practice reached approximately 3.07 million hectares. In this area, rice-crayfish co-culture accounted for approximately 56.46% of the total integrated rice-aquaculture farming and represented the dominant production model, with crayfish (*Procambarus clarkii*) as the principal cultivated species (National Aquatic Products Technology Extension Station and Chinese Society of Fisheries, 2025). In rice-crayfish systems, residual feed and feces generated during crayfish farming constitute substantial organic fertilizer inputs. Their stable organic compounds can gradually release nutrients and stimulate microbial growth and activity in integrated rice-crayfish soils (Rong et al., 2025). Paddy fields can function as intermittent wetland ecosystems and provide favorable habitats for active microbial communities and associated metabolic processes, supporting diverse and abundant microbial communities (Hou et al., 2025). Carbon (C) and nitrogen (N) are fundamental nutrients in agroecosystems. Microbial processes involved in C and N cycling, including organic carbon and organic nitrogen degradation, and greenhouse gas-related transformations, are central to sustainable agricultural development. As soil microorganisms are highly responsive to external disturbances, assessing microbial changes in intermittent wetland systems is necessary for improving rice-crayfish soil quality and promoting agricultural sustainability.

Extensive studies have been reported on straw decomposition and the associated succession of microbial communities. However, when straw is directly returned to the field in an integrated rice-aquaculture system, whether straw can be effectively decomposed, and whether it enhances paddy soil fertility remain largely unexplored. In this study, a rice-crayfish integrated system combined with straw return to paddy fields was established to examine the changes in soil fertility, enzyme activity, and microbial diversity in an intermittent wetland ecosystem. We proposed the following hypotheses: (1) Crayfish farming activities (mainly residual feed and fecal inputs) effectively promote the decomposition of rice straw. By the rice transplanting stage following the crayfish farming period (July, PF7), straw decomposition would have progressed to the late stage, with readily degradable components almost completely decomposed-coinciding with the nutrient demand of the rice crop-while the more recalcitrant organic fractions would decompose slowly throughout the rice growing season. (2) During the post-harvest field drying period (November, PF11), the drying treatment would further facilitate straw decomposition while simultaneously reducing greenhouse gas emissions potential. To test the above hypotheses, a rice-crayfish integrated system with straw return was established. Through soil physicochemical characterization, enzyme activity assays, and metagenomic sequencing, the changes in soil fertility, enzyme activities, and microbial functional profiles were systematically investigated. The specific objectives were: (1) to assess the evolution of soil fertility and the potential for straw decomposition under this model; (2) to elucidate the decomposition pathways of rice straw by analyzing the microbial mechanisms of carbon and nitrogen cycling, and to evaluate the ecological benefits of the system, including its capacity to promote straw decomposition, sustain soil fertility, and reduce greenhouse gas emissions potential. This study aims to clarify the decomposition process of straw within the integrated rice-aquaculture system, thereby providing a theoretical foundation for its wider application and promoting the efficient utilization of straw resources.

## 2 Materials and methods

### 2.1 Experiment

A rice-crayfish integrated farming field from Feixi Longguanhe Ecological Agriculture Co., Ltd. (117.34°E, 31.58°N) was selected to represent an intermittent wetland ecosystem. Within this system, a paddy field (about 1.67 ha) was used as the experimental treatment (Paddy field, PF), and the surrounding ditch, representing a prolonged flooded area, was adopted as the control group (CK). The crayfish in this study was the red swamp crayfish (*Procambarus clarkii*). The ditch occupied 9%–10% of the total paddy field area and was 1 m deep. After the rice was harvested in mid-October, the straw was shredded and spread evenly across the paddy field, after which the field was dried until the following February. During this period, 1-m-wide grass paths were created at 5-m intervals using a rotary tiller, and organic fertilizer (pigeon feces, 1.4 t ha^–1^) was applied. In early February, the field was reflooded to a water depth of 10–20 cm, whereas the ditch water depth was maintained at 0.8 m. Elodea nuttallii was planted in the rotary-tilled grass paths, and juvenile crayfish (approximately 10–20 g per individual) were subsequently released at approximately 0.075 t ha^–1^. From February to June, the juvenile crayfish were supplied with approximately 1.73 t ha^–1^ of feed containing 36%–40% protein. Water quality was regulated using mineral source potassium fulvic acid (0.06 t ha^–1^), sodium humic acid (0.0225 t ha^–1^), Bacillus, and small algae. In June, after crayfish farming ended, water was drained from the rice paddies, compound fertilizer was applied, and the fields were tilled for rice planting. The fertilizer was applied at 0.375 t ha^–1^ (N + P_2_O_5_ + K_2_O, 22-10-13). In addition, the ditch bottom soil was transferred to the paddy field after rice harvest.

### 2.2 Sample collection

Surface soil samples were collected in July 2025, after crayfish harvest and before rice transplantation (PF7), and in November 2025, post-harvest field drying period (PF11). In the paddy field, five soil samples (0-20 cm) were collected in an S-shaped pattern. In the surrounding ditch, five 0–20 cm soil samples were collected at 4–5 m intervals and designated as CK7 and CK11, respectively. The samples for microbial analysis determination were frozen at −80 °C, whereas the samples for soil component analysis were air-dried before determination.

### 2.3 Detection of physicochemical parameters of soil

Soil pH was determined using a pH meter at a 1:2.5 (w/v) solid-to-liquid ratio (Wang et al., 2007). Available potassium (AK) was extracted using neutral 1 mol/L ammonium acetate solution and quantified using flame photometry (Jiao et al., 2015). Total nitrogen (TN) was measured using the Kjeldahl method after Na_2_S_2_O_3_-HClO_4_-H_2_SO_4_ digestion (Tianjin Environmental Monitoring Center, 2015). Soil organic matter (SOM) was determined using the standard K_2_Cr_2_O_7_-H_2_SO_4_ oxidation method (Ren et al., 2006). Ammonium nitrogen (NH_4_^+^-N) was determined by the indophenol blue colorimetric method after extraction with 2 mol/L KCl solution, alkali-hydrolyzable nitrogen was measured using the alkaline hydrolysis diffusion method, and nitrate nitrogen (NO_3_^−^-N) was extracted with 2 mol/L KCl and quantified by ultraviolet spectrophotometry at 220 nm and 275 nm for background correction.

### 2.4 Enzyme activity

Enzyme activity was determined using 0.1 g of air-dried soil following the instructions of the Wilming enzyme activity kit. Protease, urease, cellulase, and sucrase activities were measured at 680, 578, 540, and 510 nm, respectively, using a SpectraMax Single Mode Reader (ABS Plus).

### 2.5 Metagenome sequencing and analysis

DNA was extracted from 0.5 g of soil using a Soil DNA Kit (Omega Bio-Tek, USA). DNA purity was measured using NanoDrop 2000, and DNA concentration was measured using TBS-380. Genomic DNA was fragmented to approximately 400 bp using a Covaris M220 ultrasonic disruptor. A paired-end (PE) library was constructed using the NEXTFLEX/Rapid DNA-Seq Kit. After 5′ phosphorylation, the library was circularized and subjected to rolling circle amplification to generate DNA nanospheres (DNBs), which were subsequently loaded onto the flow cell. Sequencing was conducted on the DNBSEQ-T7 platform.

After the sequencing data were obtained, raw reads were subjected to quality control and trimming using fastp software (version 0.20.0). Adapter sequences were removed from both the 5′ and 3′ ends, and low-quality reads were discarded according to either of two criteria: (1) length < 50 bp after trimming or (2) average Phred quality score < 20, corresponding to a base error rate > 1%. The remaining high-quality clean reads were used for downstream analyses. The de novo assembly was conducted using MEGAHIT^1^ (version 1.1.2), which was based on the succinct de Bruijn graph algorithm. Contigs with lengths ≥ 500 bp were retained as the final assembly results. The open reading frames (ORFs) in the assembled contigs were predicted using Prodigal^2^. Genes with nucleic acid lengths ≥ 200 bp were retained and translated into amino acid sequences. The predicted gene sequences from all samples were clustered using CD-HIT^3^ (version 4.6.1), with the parameters set to 95% identity and 90% coverage. Within each cluster, the longest gene sequence was selected as the representative sequence to construct a non-redundant (NR) gene set. High-quality reads from each sample were aligned to the NR gene set using bowtie2^4^ at 95% identity, and gene abundance information was calculated for the corresponding samples. Microbial metabolic potential was quantified as the normalized abundance of functional genes, calculated as transcripts per million (TPM). The amino acid sequences in the NR gene set were aligned against the NCBI NR database using Diamond^5^ (version 0.8.35), with the BLASTP e-value set to 1e^–5^. Taxonomic annotations were obtained from the corresponding taxonomic information database of the NR library. Species-level abundance was calculated by summing the abundance of genes assigned to each taxon. Functional annotation was performed by aligning amino acid sequences against the KEGG database (version 94.2) using Diamond^6^ (version 0.8.35), with BLASTP parameters set to e-value ≤ 1e^–5^. The KEGG functions corresponding to the genes were obtained, and the abundance of each functional category was calculated by summing the gene abundances corresponding to KO, Pathway, EC, and Module. Carbohydrate-active enzyme (CAZyme) annotation was performed using hmmscan against the CAZy database, with an e-value ≤ 1e^–5^. CAZyme family annotations were retrieved, and the abundance of each CAZyme family was calculated by summing the abundance of genes annotated to that family. The raw sequencing data have been deposited in the NCBI bioproject under accession number PRJNA1528484.

### 2.6 Statistical analysis

All results are presented as mean ± standard deviation, which were calculated using Microsoft Excel 2022, and graphics were generated using Adobe Illustrator 2022. For C-degradation subclasses, a one-KO-one-subclass rule was applied to avoid double counting: KOs matching multiple subclasses were assigned by functional specificity (amino sugars and nucleotide sugars > pectin and uronic acids > starch and sucrose > fatty acids and sterols > aromatic compounds > amino acids). The N cycle required no such rule, as each KO maps to a single pathway. Redundancy analysis (RDA) was performed and visualized using Canoco 4.5. Partial Least Squares Path Modeling (PLS-PM), Mantel’s test, and network analysis were performed and visualized using R software (version 4.5.2).

## 3 Results

### 3.1 Physicochemical properties and enzyme activity of soil

As shown in Table 1, all measured soil fertility indicators (K, N, C) were higher in PF7 than in CK7, suggesting that straw decomposition released N and K, and promoted SOM accumulation, thereby enhancing soil fertility. In November, however, N and K fertilizers in PF11 were lower than in PF7, with the exception of SOM. Despite N and K inputs from crayfish farming, straw return, and compound fertilizer application during the rice growing season, those contents declined due to continuous uptake by the rice crop and drainage losses. In contrast, SOM showed a slight increase, consistent with the sustained carbon input from gradual decomposition of straw residue and organic matter.

**TABLE 1:** The physicochemical properties and enzyme activity in PF and CK.

Parameter	CK7	PF7	CK11	PF11
pH	5.67 ± 0.52	5.09 ± 0.19	5.86 ± 0.31	5.46 ± 0.3
SM (%)	29.90 ± 0.04	28.53 ± 0.04	28.67 ± 0.04	25.72 ± 0.04
AK (mg kg^–1^)	149 ± 3.46	167 ± 17.32	144 ± 6	65.2 ± 3.29
TN (g kg^–1^)	1.56 ± 0.12	1.67 ± 0.07	0.42 ± 0.1	1.35 ± 0.09
NH_4_^+^-N (mg kg^–1^)	15.34 ± 1.02	16.74 ± 4.27	8.35 ± 1.60	7.22 ± 2.22
Alkaline hydrolyzable N (mg kg^–1^)	104.13 ± 10.64	136.36 ± 15.43	46.7 ± 3.73	105.8 ± 20.68
NO_3_^–^-N (mg kg^–1^)	23.05 ± 2.53	34.68 ± 4.77	3.72 ± 0.64	7.29 ± 1.18
SOM (g kg^–1^)	21.5 ± 0.92	23.63 ± 0.64	16.79 ± 1.6	23.87 ± 2.51
Protease (mg d^–1^ g^–1^)	0.39 ± 0.02	0.38 ± 0.02	0.10 ± 0.00	0.24 ± 0.01
Urease (μg d^–1^ g^–1^)	942.64 ± 27.11	1264.95 ± 56.14	918.73 ± 28.28	1105.32 ± 26.72
Cellulase (μg d^–1^ g^–1^)	28.16 ± 0.56	68.50 ± 1.10	42.42 ± 3.26	61.33 ± 8.43
Sucrase (mg d^–1^ g^–1^)	3.62 ± 0.12	3.63 ± 0.16	1.24 ± 0.04	3.11 ± 0.12

SM, soil moisture; AK, available potassium; TN, total nitrogen; SOM, soil organic matter.

Soil nutrient mineralization and organic matter decomposition are closely regulated by soil enzyme activity, whose activities are widely used as indicators of soil quality and biological activity (Zhang et al., 2025). We therefore compared the activities of four enzymes (protease, urease, cellulase and sucrase) involved in C and N cycling between the paddy field (PF) and the surrounding ditch (CK) (Table 1).

In July, PF7 exhibited higher urease and cellulase activities than CK7, with increases of 34% and 143%, respectively. In contrast, protease and sucrase activities showed similar levels between the two treatments (Table 1). In November, the activities of protease, urease, cellulase, and sucrase in PF11 were all higher than those in CK11, which could be attributed to the transfer of organic matter-rich sediment from the surrounding ditch to the paddy field. However, compared with PF7, the activities of protease, urease, and sucrase in PF11 declined, whereas cellulase activity remained similar.

### 3.2 Microbial diversity and abundance

To further clarify the soil microbial community structure, metabolic pathways, and straw decomposition processes in paddy fields under the rice-aquaculture co-culture system, soil microbial communities were analyzed using metagenomic sequencing.

The changes in microbial community composition at the phylum level are shown in Figure 1A, and the corresponding abundances are presented in Supplementary Table 1. These phyla accounted for 79.98%–83.40% of the total abundance. Across all groups, Pseudomonadota was the most abundant bacterial phylum, comprising 21.91%–32.02% of the community.

**FIGURE 1:**
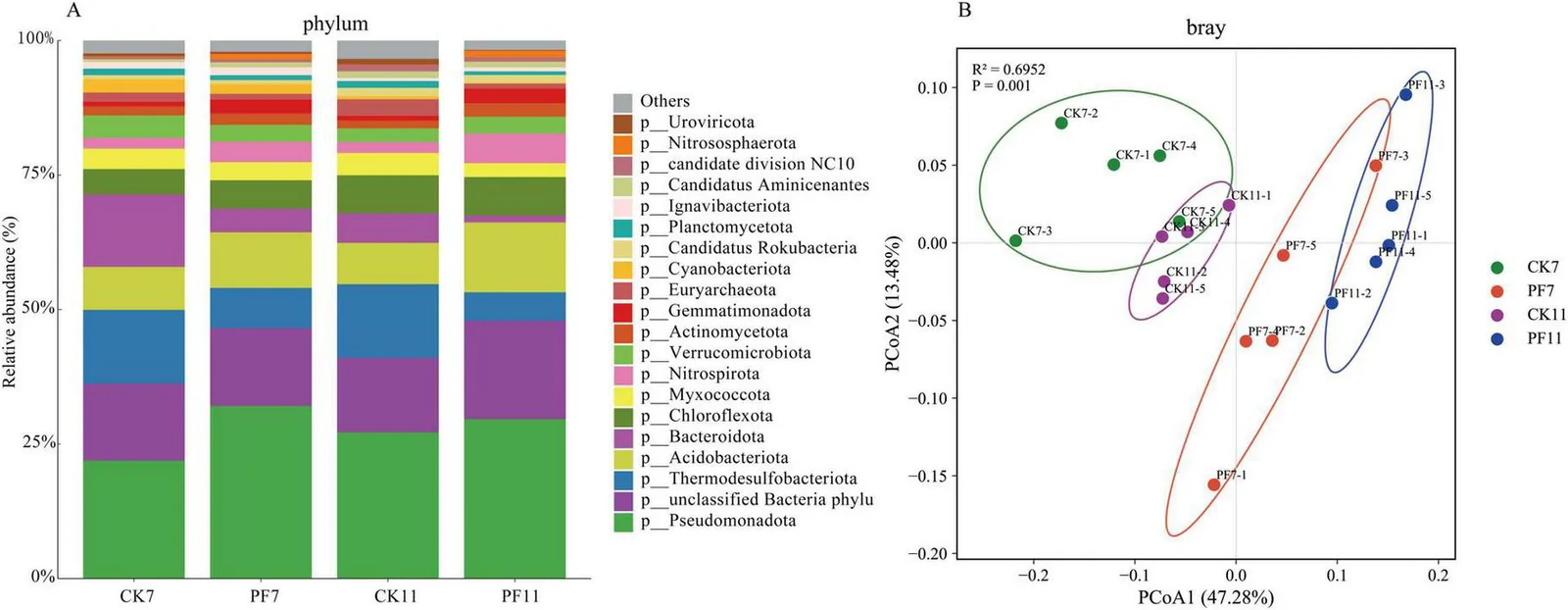
Composition of soil microbial communities. **(A)** Relative abundance of microbial communities at the phylum level. **(B)** Principal Coordinates Analysis (PCoA) of metagenomic results at the phylum level.

Several phyla exhibited marked differences in relative abundance between PF7 and CK7. The abundance of Bacteroidota in PF7 was 0.35-fold that of CK7, indicating a suppression of this phylum in the straw amended paddy soil. In contrast, Nitrososphaerota and Gemmatimonadota were 7.60- and 2.74-fold more abundant in PF7 than in CK7, respectively, the corresponding values for PF11 versus CK11 were 10.57- and 4.46-fold, suggesting an enrichment of ammonia oxidizing archaea and organic degrading bacteria in response to straw return and organic fertilizer inputs. By November, these differences between PF11 and CK11 had further widened. Additionally, the abundances of Euryarchaeota and Thermodesulfobacteriota in PF11 decreased to 0.23-fold and 0.35-fold of the corresponding CK11 levels. In the case of Cyanobacteriota, a distinct response pattern was observed. Its abundance in PF7 was higher than in CK7, while the most pronounced change occurred from PF7 to PF11, with PF11 being only 5.7% of PF7 level. This pattern was mainly associated with differences in water depth. In PF7, shallow residual water and favorable light penetration supported photosynthesis. During the sun-drying stage of PF11, the extremely limited water availability sharply reduced Cyanobacteriota abundance. Conversely, Cyanobacteriota remained relatively abundant in the adjacent ditch, where sustained water cover, together with nutrient inputs from residual feed, crayfish feces and field drainage, likely maintained favorable conditions for cyanobacterial growth.

The PCoA analysis based on Bray-Curtis is shown in Figure 1B. PCoA1 and PCoA2 explained 47.28% and 13.48% of the variances, respectively. Clear separation was observed between PF and CK groups in both July and November. Partial overlap existed between PF7 and PF11 samples, and a similar overlapping pattern was also found within the CK group. The genus-level taxonomic profiles of the microbial communities and PCoA analysis are presented in Supplementary Figure 1, in which CK7 and CK11 were well distinguished.

### 3.3 C cycling and key functional genes

KEGG level 3 annotation was used to identify microbial functional genes associated with soil organic carbon decomposition. Based on this annotation, C cycling was divided into four major metabolic pathways: C degradation, organic acid metabolism, C fixation, and CH_4_ metabolism (Figure 2A). Across all treatments, C degradation was the dominant component of C cycle, representing the largest proportion of total C cycling potential, at 78.37%–79.92% (Figure 2A).

**FIGURE 2:**
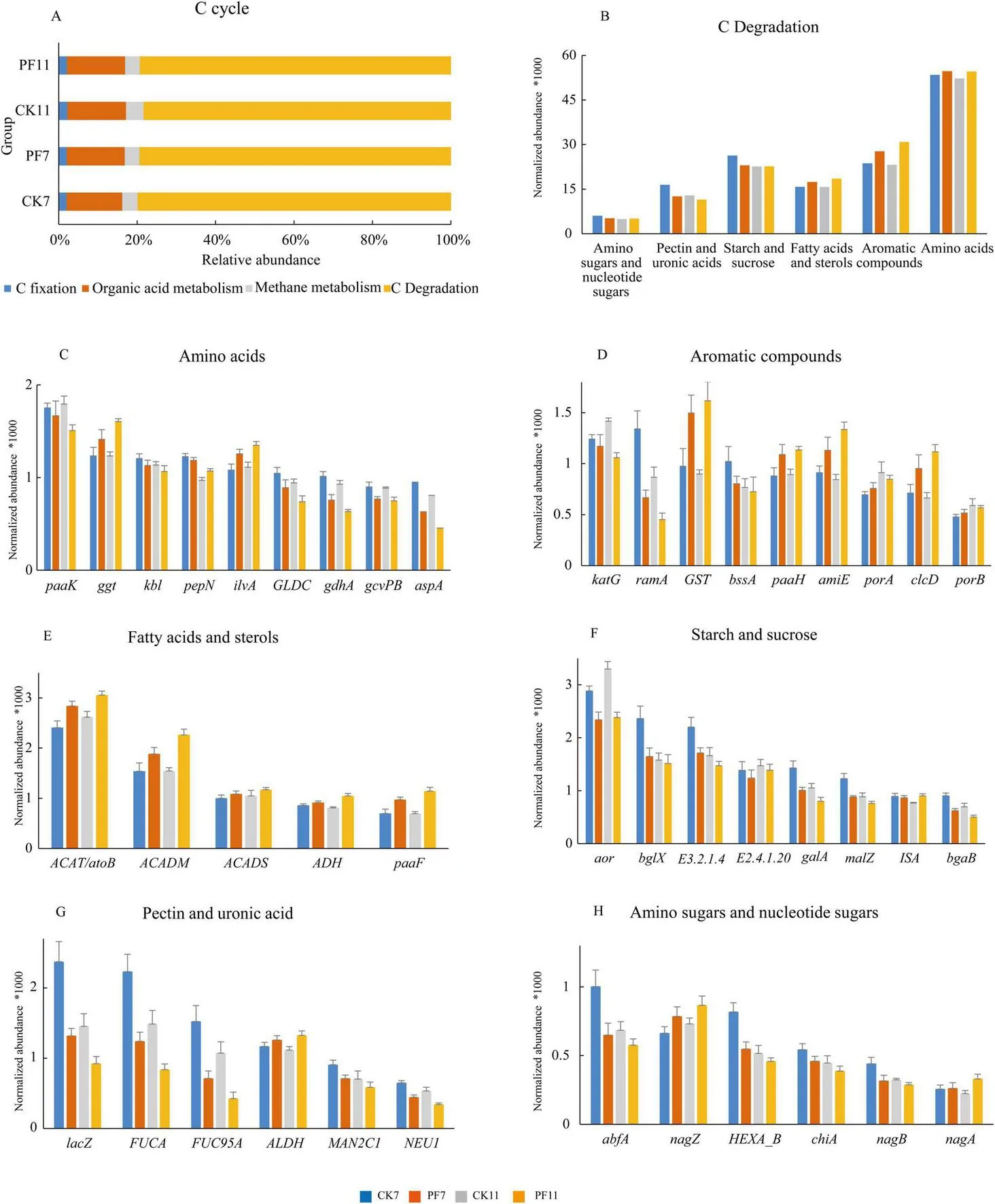
Abundance of functional genes related to C cycles. **(A)** Relative abundance of functional genes related to C cycle. **(B)** Normalized abundance of carbon degradation pathways. Normalized abundance of the main functional genes involved in the degradation of **(C)** amino acids, **(D)** aromatic compounds, **(E)** fatty acids and sterols, **(F)** starch and sucrose, **(G)** pectin and uronic acid, and **(H)** amino sugars and nucleotide sugars.

C degradation mainly included the breakdown of amino acids, aromatic compounds, fatty acids and sterols, starch and sucrose, pectin and uronic acids, amino sugars and nucleotide sugar (Figure 2B). Amino acid degradation was overwhelmingly dominant, contributing 37.71%–39.73% of the C degradation potential across treatments, which we attribute to the substantial organic inputs from crayfish farming (i.e., residual feed and feces) that sustained a continuous supply of proteinaceous substrates for the microbial community. Notably, the abundance of aromatic compound degradation genes ranked second and was consistently higher in PF than in CK soils, indicating that straw return enhanced the genetic potential for degrading recalcitrant aromatic structures derived from lignin and other refractory straw constituents. In parallel, the abundance of fatty acid and sterol degradation was enriched in PF, whereas pectin and uronic acid degradation genes declined, consistent with the progressive exhaustion of readily degradable pectin-like substrates in straw-amended soils. The dominance of amino acid, fatty acid, and aromatic compound degradation in PF treatments was consistent with the lower soil pH (Table 1), as these catabolic processes release NH_4_^+^-N, organic acids, and phenolic intermediates that may collectively contribute to soil acidification.

The abundances of the corresponding functional genes are shown in Figures 2C–H. It could be clearly seen that genes about aromatic compound degradation exhibited mixed responses (Figure 2D), with both up- and downregulation observed. Specifically, the abundance of *GST*, *paaH*, *amiE* and *clcD* were higher in the PF, whereas *katG* and *ramA* were markedly decreased. The higher abundance of *paaH*, *amiE*, and *clcD*, which are directly involved in aromatic ring cleavage and downstream intermediate transformation, suggests that recalcitrant aromatic compounds potentially derived from straw lignocellulose were being actively degraded by the microbial community.

The CAZyme composition and diversity were analyzed to indicate the microbial genetic potential for organic C decomposition and biosynthesis. Among all annotated carbohydrate-active enzymes in the consortium, GHs and GTs were the two main groups, accounting for 44.32-53.03% and 37.88-46.19% of total CAZyme gene abundance, respectively (Figure 3A). Normally, CAZymes are categorized into six classes. However, in this system, the abundance of AA was extremely low and almost negligible; therefore, only five classes are displayed in Figure 3A. These enzymes play key roles in cleaving polymeric carbohydrate substrates. GH5, GH31, and GH29 showed consistent declining trends, with lower abundances in PF7 than in CK7 and further reductions in PF11 (Figure 3B). These families mainly consist of beta-glucosidases, endo-β-1,4-glucanases, alpha-glucosidases, and alpha-L-fucosidases, which are involved in cellulose, hemicellulose, and starch hydrolysis (Pan et al., 2021; Jose et al., 2017). GH94 reached its highest level in PF11, which could catalyze the reversible phosphorolysis of cellobiose into α-glucose 1-phosphate and glucose (Jose et al., 2017). These results suggest that straw decomposition had entered a late stage by July, with readily degradable components largely depleted and aromatic compounds accumulating as predominant residues. The elevated GH94 abundance in PF11 further indicates that cellulose degradation continued through the growing season, likely targeting the remaining cellulosic fractions, while the intensified decomposition of aromatic compounds in November was consistent with the enrichment of aromatic ring compound degradation pathways.

**FIGURE 3:**
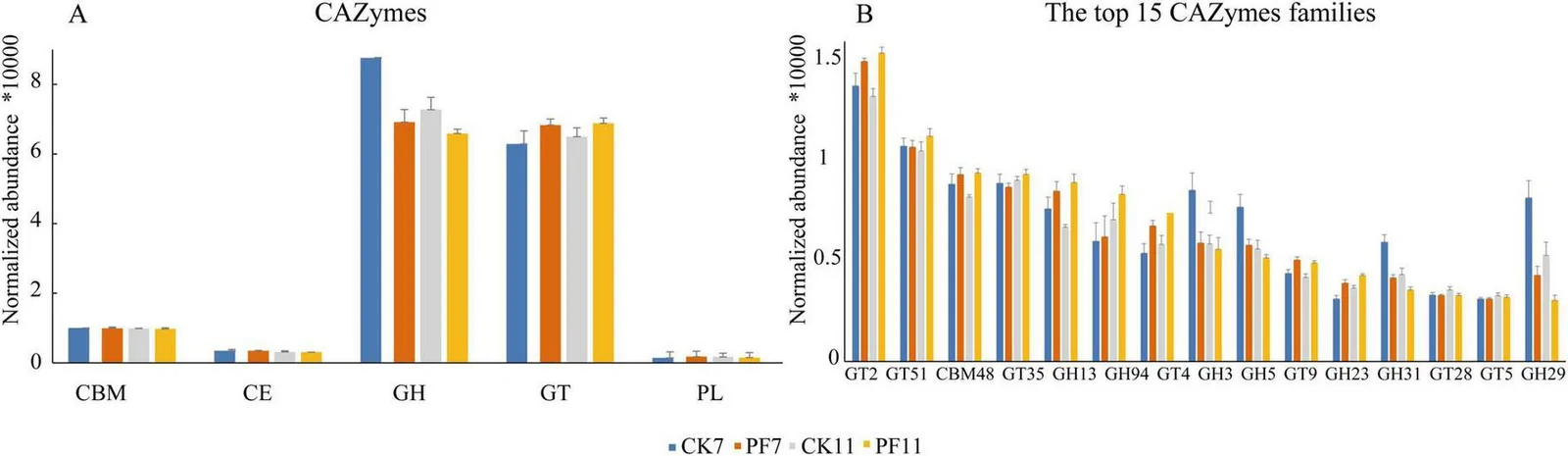
Profiles of CAZyme-encoding functional genes. **(A)** Normalized Abundance of CAZyme-encoding functional genes. **(B)** Normalized Abundance of the top 15 CAZyme families.

Figures 4A, B show the metabolic pathway of CH_4_, and the corresponding main functional genes are shown in Figures 4C–F. Among the CH_4_ metabolic pathways, acetoclastic methanogenesis was the dominant pathway in both PF7 and PF11, representing 43.67% and 46.74% of methane metabolism pathway abundance, respectively (Figures 4A, B). As acetic acid serves as a key intermediate in anaerobic organic matter decomposition, this dominance suggested that both the paddy field and the ditch contained substantial organic matter, consistent with the common characteristics of paddy soils and freshwater sediments (Hou et al., 2025). At the gene level, *mcrA*, a key gene involved in methanogenesis, was lower in the PF than the CK (Figure 4C), and acs, another key gene in acetoclastic methanogenesis, showed comparable levels between PF and CK (Figure 4E). In contrast, the gene *pmoA*, driving CH_4_ conversion to carbon dioxide, showed higher abundance in PF than in CK. Moreover, compared with PF7, methanogenesis related genes in PF11 showed a decreasing trend (Figure 4A). The lower *mcrA* abundance in PF suggest a shift toward reduced net methane emission potential. This pattern was consistent with the reduced greenhouse gas emission potential observed under this integrated system.

**FIGURE 4:**
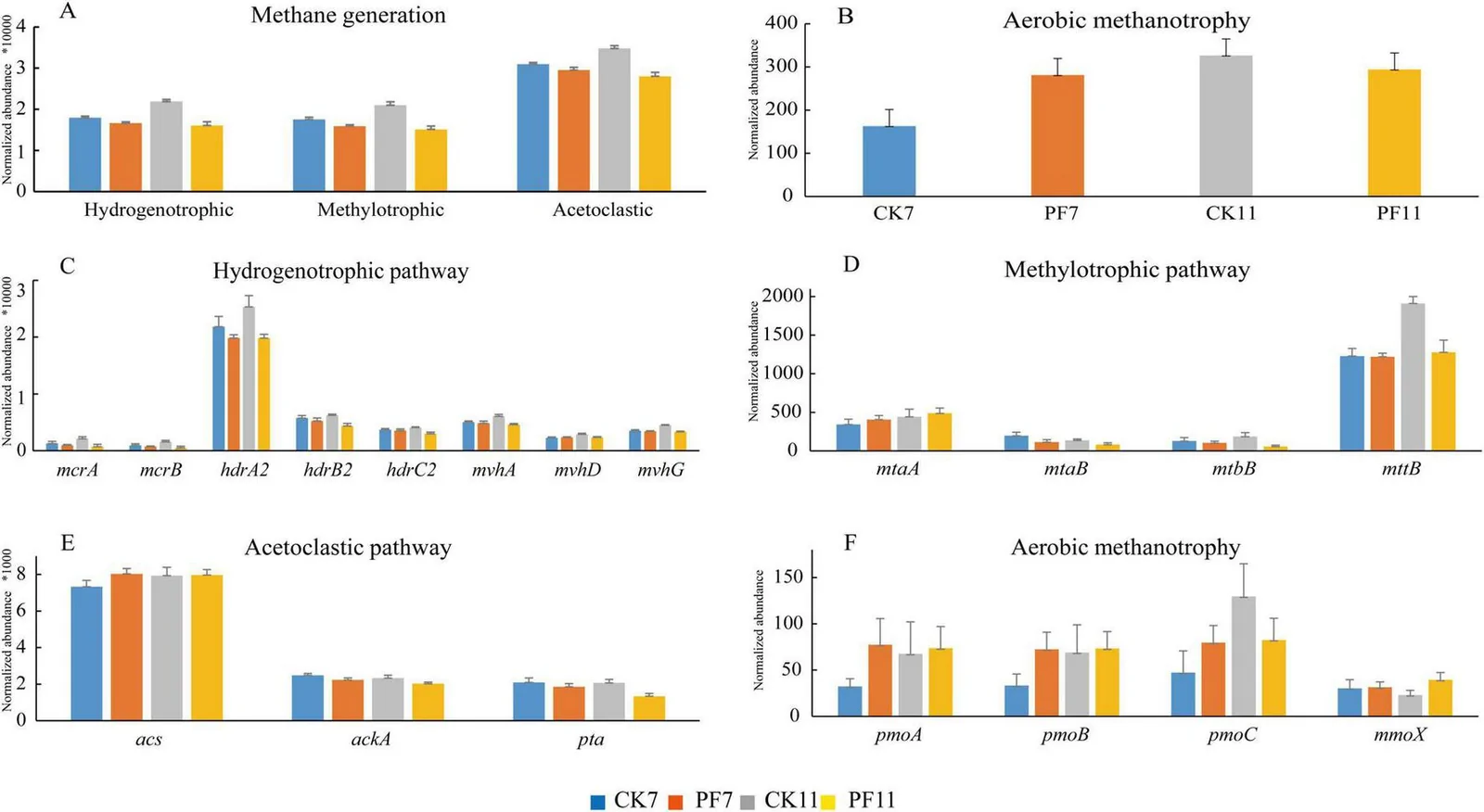
Abundance of functional genes related to methane metabolism. Normalized abundance of **(A)** methane generation pathway, and **(B)** aerobic methanotrophy pathway. Normalized abundance of the main functional genes related to **(C)** hydrogenotrophic pathway, **(D)** methylotrophic pathways, **(E)** acetoclastic pathways, and **(F)** aerobic methanotrophy.

### 3.4 N cycling and key functional genes

Functional genes associated with N cycling were assigned to six metabolic pathways: N fixation, nitrification, denitrification, dissimilatory nitrate reduction to ammonium (DNRA), assimilatory nitrate reduction to ammonium (ANRA) and organic N metabolism (Figure 5A). The corresponding main functional genes are shown in Figures 5B–G. Notably, organic N metabolism contained the highest proportion of genes, accounting for 81.63–84.14% of all N cycling genes across treatments. Genes related to nitrification, denitrification, N fixation, DNRA, and ANRA followed in abundance. At the pathway level, organic N metabolism, N fixation and DNRA were lower in PF than in CK. In contrast, ANRA, nitrification and denitrification were higher in PF. At the functional gene level, the abundance of *glnA*, *gltB/gltD*, *gdhA*, *ureC* (Organic N metabolism), *nifH/nifD*/*nifK* (N fixation) and *nrfA/nrfH* and *nirB* (DNRA) were obviously lower in PF (Figures 5B, C, E). In contrast, *nxrA* (nitrification), *nasA/nasB* and *nirA* (ANRA) showed higher abundance in PF than in CK (Figures 5D, G).

**FIGURE 5:**
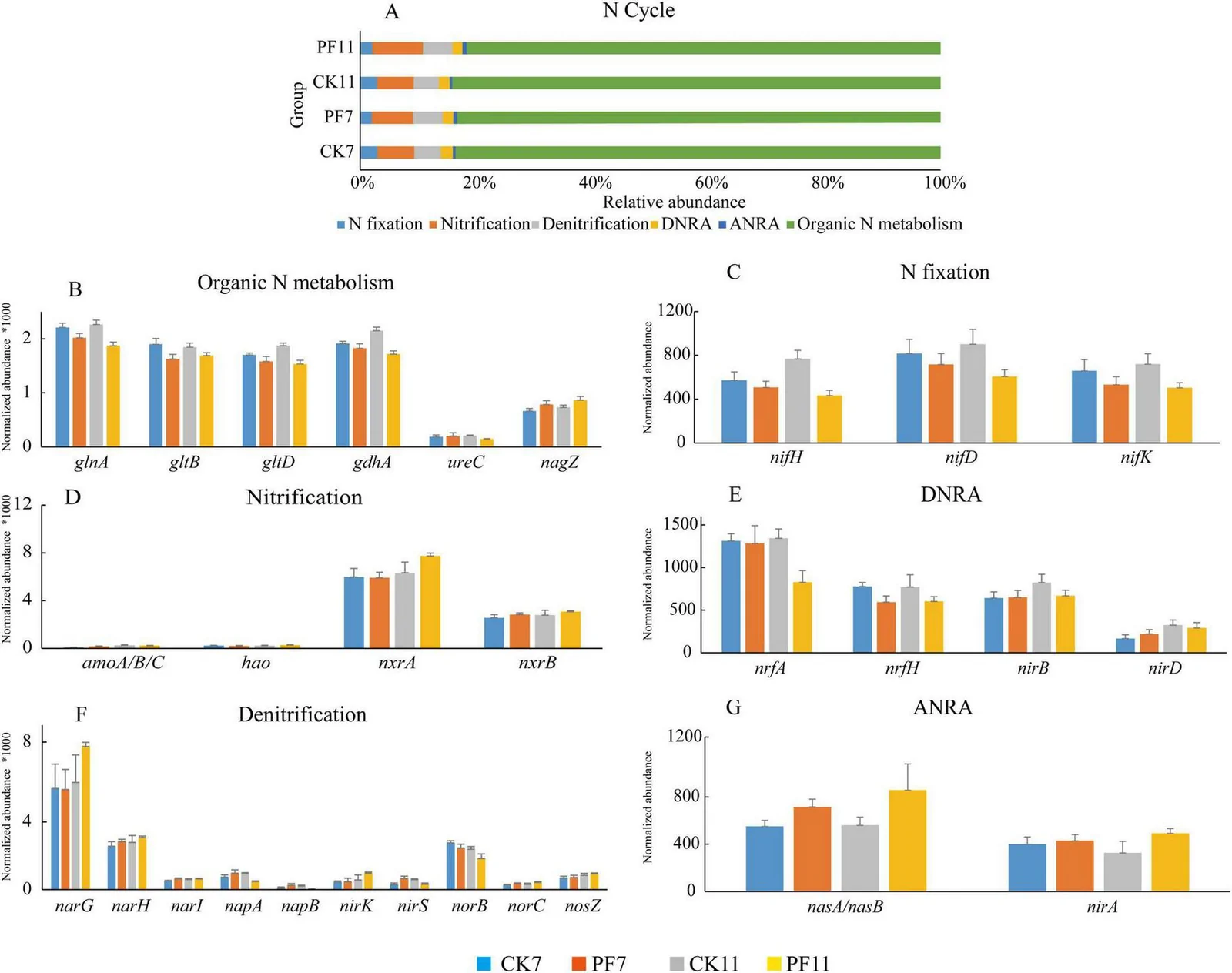
Abundance of functional genes related to N cycle. **(A)** Relative abundance of functional genes related to N cycle. Normalized abundance of the main functional genes related to **(B)** organic N metabolism, **(C)** N fixation, **(D)** nitrification, **(E)** dissimilatory nitrate reduction to ammonium (DNRA), **(F)** denitrification, and **(G)** assimilatory nitrate reduction to ammonium (ANRA).

Denitrification is the microbial process by which nitrate is reduced to nitrogen gases (N_2_ or N_2_O), which are then released into the atmosphere. Total denitrification pathway abundance was higher in PF (Figure 5A). However, the responses of individual functional genes within this pathway were not entirely consistent with the pathway-level trend (Figure 5F). The nitrate reductase gene *narG* was markedly more abundant in PF11 than in CK11, indicating sustained enhancement of nitrate reduction potential. The nitric oxide reductase gene *norB* was lower in PF, with the lowest level observed in PF11. The nitrite reductase genes *nirK* and *nirS* showed opposing trends: *nirK* increased in PF11, whereas *nirS* decreased. The nitrous oxide reductase gene *nosZ* showed higher abundance in PF11 than in CK11. The lower *norB* but higher *nosZ* in PF11 indicate a reduced N_2_O emission potential.

### 3.5 Relationships among soil properties, enzyme activities, microbial communities, and C-cycling and N-cycling genes

As soil nutrients influence microbial growth, redundancy analysis (RDA) was used to assess the relationships between microbial community composition and soil environmental variables (Figure 6). The first and second RDA axes explained 61.60% and 10.70% of the total variation in the microbial community structure, respectively, yielding a cumulative explanatory power of 72.30%. However, the permutation test was not significant (*p* = 0.2123). These results suggest that the selected environmental variables explained a substantial proportion of the variation but did not reach statistical significance.

**FIGURE 6:**
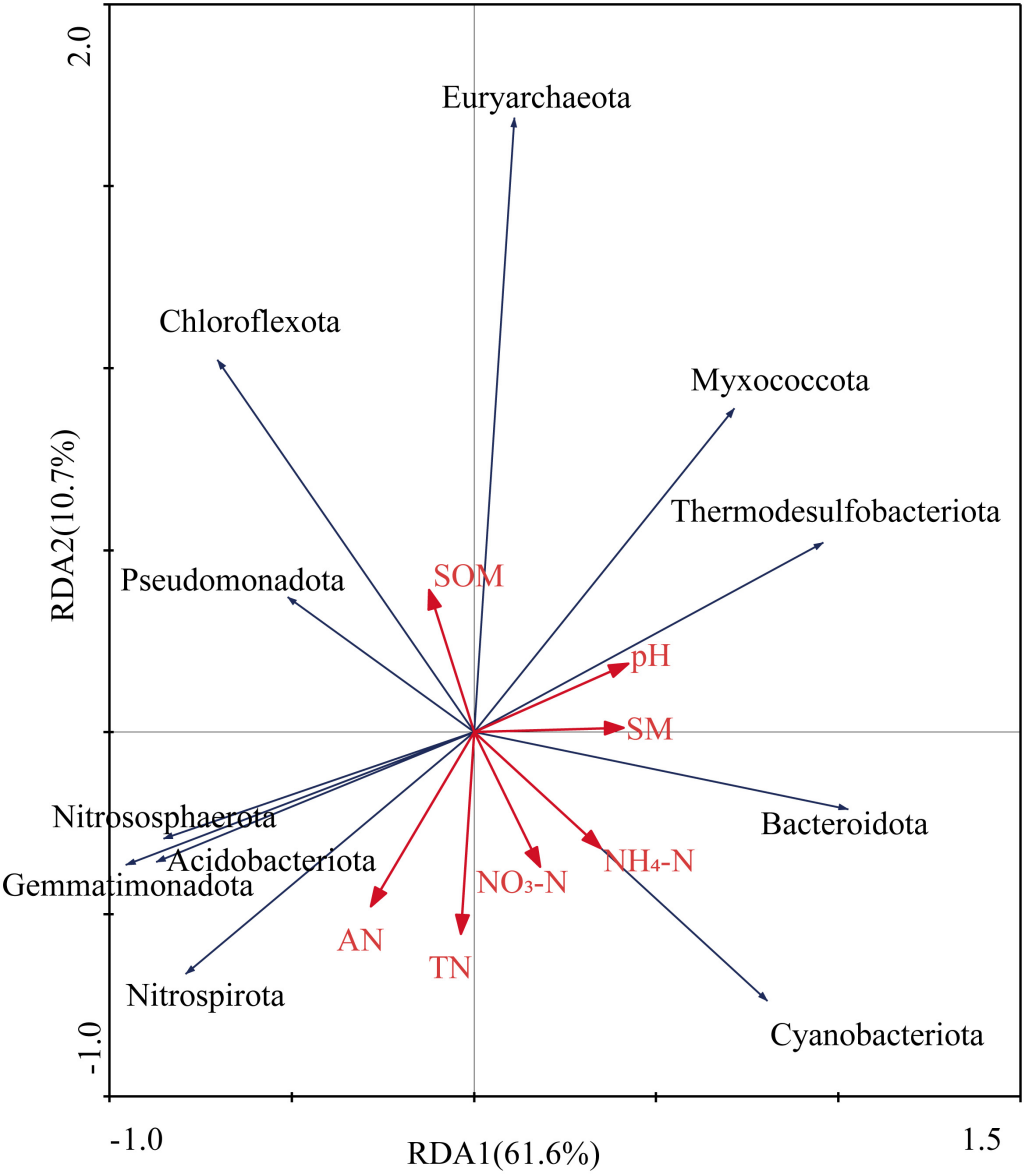
Redundancy analysis (RDA) of microbial communities at the phylum level and soil environmental factors.

Among the measured soil physicochemical properties, soil moisture (SM) exhibited positive association trends with Bacteroidota, Thermodesulfobacteriota, Myxococcota and Cyanobacteriota. Among various nitrogen-form indices, alkaline-hydrolyzable nitrogen (AN) showed the strongest correlation with Nitrospirota and Nitrososphaerota, whereas ammonium nitrogen (NH_4_^+^-N) displayed the weakest linkage. Soil organic matter (SOM) was positively associated with Chloroflexota, Euryarchaeota, Pseudomonadota and Myxococcota. Notably, among all measured soil physicochemical variables, SOM exhibited the strongest positive linear trend with Euryarchaeota, whereas total nitrogen (TN) showed a negative linear trend with this archaeal taxon. No distinct linear relationship was detected between SM and Euryarchaeota.

The co-occurrence network of functional genes involved in C and N cycling is shown in Figure 7. This network consisted of 67 nodes and 411 edges, with an average degree of 12.27, a graph density of 0.186, and a clustering coefficient of 0.452 (Figure 7A). Among the top 15 genes ranked by degree centrality, 10 were associated with C cycling and 5 with N cycling (Figure 7B). This pattern demonstrates the central role of C cycling in microbial networks and indicates that C metabolism is the primary driver of microbial community structure in this paddy field system. In total, 57.96% of the C- and N-cycling genes demonstrated positive co-occurrence relationships.

**FIGURE 7:**
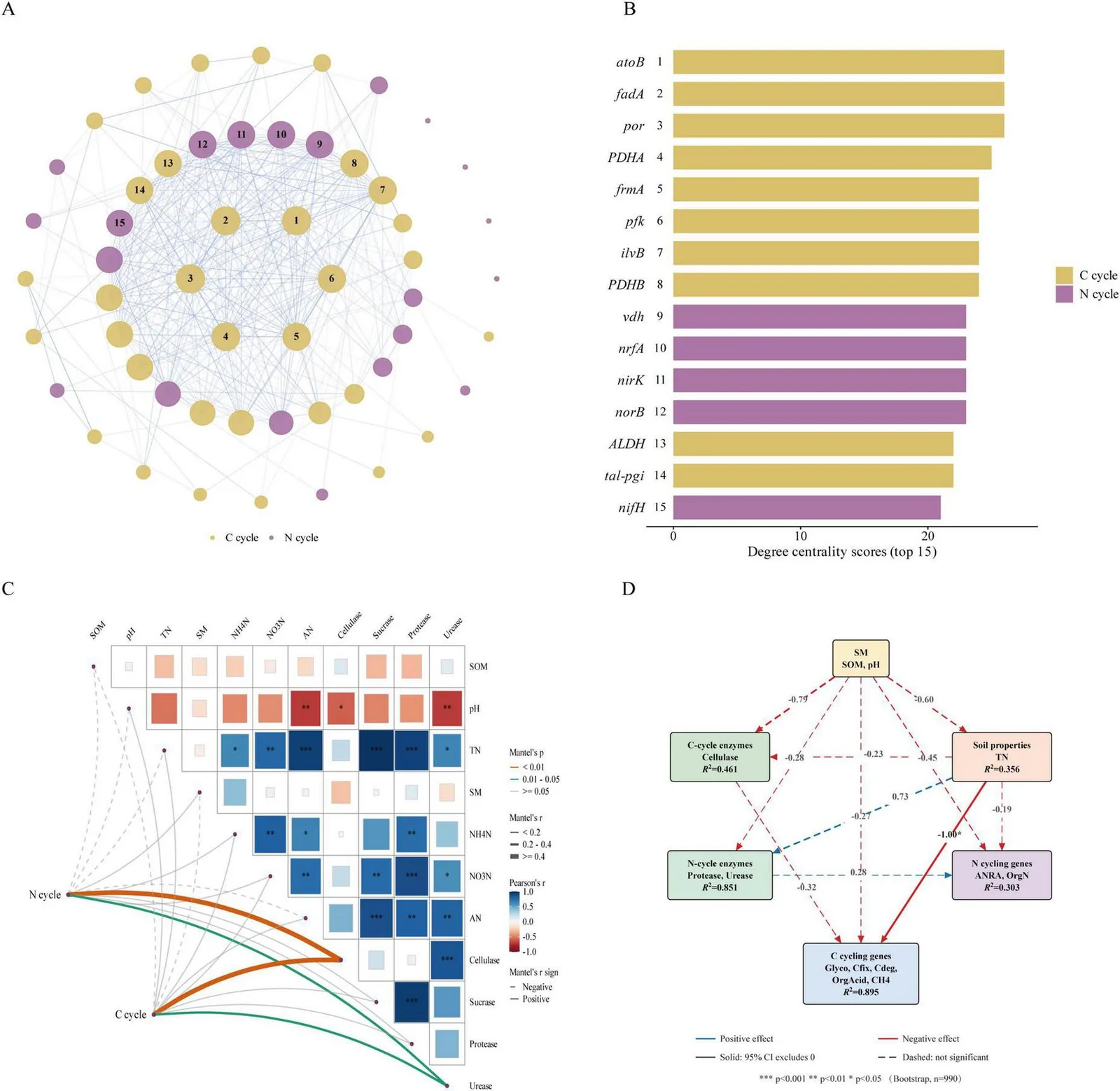
Relationship between soil properties and C and N cycling genes. **(A)** Network analysis shows interactions between C and N cycling genes. **(B)** Degree scores for the top 15 genes in the networks. **(C)** Mantel’s test about the relationships between functional genes involved in C and N cycling and soil properties and enzyme activity. **(D)** Partial Least Squares Path Modeling (PLS-PM) showing how amendments application affected the soil C and N cycling genes.

To further evaluate the relationships among soil physicochemical properties, extracellular enzyme activities, and microbial functional genes, Mantel’s tests and Partial Least Squares Path Modeling (PLS-PM) were applied (Figures 7C, D). Mantel-based network correlation revealed that cellulase activity was strongly correlated with both C- and N-cycling genes, while urease activity was also positively correlated with both gene sets. PLS-PM further indicated that nearly all of hypothesized direct pathways were non-significant. TN showed a positive trend with N-cycle enzymes (Protease and Urease) (path coefficient 0.73), while it showed a negative trend with C cycling genes (path coefficient −1.00).

## 4 Discussion

### 4.1 Physicochemical properties and enzyme activities of soil

In the integrated system, rice straw was shredded and returned to the field after rice harvest. Following an approximately 1-month drying period, organic fertilizer was applied, water was introduced for crayfish farming from February to July, and rice was planted and harvested. Through this sequence, the growth periods of crayfish and rice were effectively integrated. This model helped establish soil environmental conditions suitable for rice growth and yield improvement (Qi and Zhu, 2026). In this model, the more pronounced differences were largely associated with the transfer of organic matter-rich sediment from the ditch to the paddy field during rice cultivation, a routine agricultural practice in this integrated system.

Straw contains substantial amounts of C, N, K and other elements, and K is released rapidly during decomposition. Li et al. (2014) reported that after 90 d, the release rate of K reached 99%, whereas those of N and C were 68% and 78%, respectively. Accordingly, the soil AK content in July was higher in the paddy field (PF7) than in the ditch (CK7). By November, AK in PF11 was remarkably lower than that in CK11. This decrease was mainly attributed to potassium uptake by rice plants during the cropping season. A similar trend was observed for other N fertilizer, indicating continuous N consumption by rice. In contrast, SOM in PF11 remained comparable to PF7, mainly due to the gradual decomposition of straw residues and organic materials. In Chinese paddy fields, the average contents of SOM and TN are 21.23 g/kg and 1.98 g/kg, respectively. In integrated rice-crayfish farming, crayfish cultivation generates large amounts of high-quality organic matter and organic nitrogen fertilizers. In this integrated system, SOM was comparable to, or slightly higher than, the national average, whereas TN content in PF remained lower than the national average.

In general, enzyme activities that catalyze the decomposition of soluble saccharides often peak at the initial stage of decomposition, followed by cellulases, while oxidases typically reach their maximum activity at the late stage (Zhou et al., 2021). In the straw return coupled with rice-crayfish system, this successional pattern was clearly reflected in the enzyme activity profiles. In July, protease and sucrase activities, which are more closely associated with soluble and proteinaceous substrates-showed similar levels between PF7 and CK7, whereas cellulase and urease activities were higher in PF7. This result indicated a clear substrate specific response pattern. Since both PF7 and CK7 received substantial organic fertilizer inputs from crayfish farming (i.e., residual feed and feces), protease and sucrase activities did not show notable differences between the two treatments. Zhao et al. (2025a) previously reported that the combination of straw and organic fertilizer decreased urease activity, which they attributed to insufficient carbon supply or excessive amendment rates. In contrast, in the present integrated rice-crayfish system, SOM was above the national paddy soil average, microbial activity remained consistently elevated, and the application rates of straw and organic fertilizer did not exceed optimal thresholds, as supported by the sustained activities of cellulase and urease observed in PF7. The elevated cellulase and urease activities in PF7, together with the unresponsive protease and sucrase activities, indicated that the readily degradable soluble sugars and proteinaceous components in straw had been largely consumed, and that cellulose degradation had entered its peak phase, advancing straw decomposition to the middle to late stage. During the rice growing season, organic matter-rich sediment from the ditch was transferred to the paddy field, reducing organic matter availability in the ditch and consequently leading to lower enzyme activities in CK11 than in PF11. In addition, with the exception of cellulase, the activities of the other enzymes declined, suggesting that cellulose in the straw sustained continuous and intensive enzymatic hydrolysis throughout the growing season, while other readily degradable substrates had been largely exhausted.

Enzyme activity variation was shaped jointly by microbial and plant activity, with differences in root exudates, rhizodeposition, and microbial communities contributing to the observed patterns. Functional microbes use these enzymes to decompose organic matter and maintain nutrient cycling and ecological stability. Mantel’s test showed that the activities of cellulase and urease were positively correlated with both C- and N-cycling genes. This relationship may be a reflection of resource-driven microbial succession, where increased enzyme activity accelerates substrate depletion and drives deterministic shifts in microbial community structure.

### 4.2 Microbial diversity

Straw return has been reported to be an effective approach for agricultural waste utilization because it supplies organic matter, enhances soil nutrient availability, and promotes microbial diversity (Hua et al., 2024; Wu et al., 2026).

At the phylum level, Pseudomonadota accounted for the largest proportion. Its involvement in the C cycle suggests that these dominant taxa may function as generalist species with diverse ecological roles, consistent with the eutrophic characteristics (Zhang et al., 2026). Furthermore, the RDA results showed that Pseudomonadota were positively correlated with SOM.

The structure and composition of soil microbial communities exhibited marked variations across different sampling times and locations, with Euryarchaeota showing the most pronounced changes. This phylum is primarily composed of methanogenic archaea (Guo et al., 2019). The RDA ordination (Figure 6) revealed that Euryarchaeota exhibited the longest arrow among all microbial phyla, identifying it as the core microbial group distinguishing the straw return-integrated rice-crayfish system (PF) from the conventional control (CK). Overall correlation patterns indicated that the relative abundance of Euryarchaeota was positively correlated with SOM, but negatively correlated with various soil nitrogen fractions. In July (PF7), straw return increased SOM content to 23.63 g/kg in PF7, compared with 21.50 g/kg in CK7. However, the relative abundance of Euryarchaeota in PF7 was only 0.80-fold that of CK7, which contradicted the observed positive correlation between SOM and Euryarchaeota. This discrepancy can be attributed to the fact that nitrogen release during the early stage of straw decomposition outpaced organic matter decomposition, remarkably elevating soil nitrogen availability in PF7. The higher nitrogen levels subsequently decreased the enrichment of methanogenic Euryarchaeota. By November (PF11), Euryarchaeota abundance in PF11 further decreased to 0.23-fold of CK11 levels. This substantial decline was driven by the combined effects of enhanced soil aeration and intensified nitrogen competition. The decreased moisture content increased ventilation within the substrate, further suppressing the relative abundance of Euryarchaeota. Concurrently, the transfer of organic matter from the ditch to the paddy field in the rice-crayfish system further widened the nitrogen content gap between PF11 (TN 1.35 g/kg) and CK11 (TN 0.42 g/kg). Given the negative correlation between nitrogen content and methanogenic archaeal abundance, the nitrogen-enriched soil environment further reduced Euryarchaeota relative abundance.

### 4.3 C/N cycling

Organic fertilizer coupled with straw can effectively promote straw decomposition. Yang et al. (2023) reported that the straw decomposition was maximized when irrigation was maintained at the maximum field water-holding capacity together with organic fertilizer application. In the system constructed, the paddy soil was continuously flooded during the crayfish culture period. Based on soil fertility properties and metagenomic sequencing results, straw decomposition entered the late stage in July after crayfish harvest and before rice transplanting. After rice harvesting in November, the decomposition of the straw returned in the previous season reached the final decay stage.

Regarding organic carbon degradation, the dominant pathways included the breakdown of fatty acids, aromatic ring compounds, and amino acids. Notably, typical straw decomposition products such as cellulose and hemicellulose were not detected as major degradation pathways in this study. The fatty acid and amino acid degradation pathways were likely associated with the decomposition of organic matter derived from crayfish farming (i.e., residual feed and feces), whereas the degradation of aromatic ring compounds is characteristic of the later stages of lignocellulose decomposition, reflecting the advanced progression of straw degradation. In the CAZyme profile, GH94 mediating alpha-glucan hydrolysis and disaccharide/oligosaccharide phosphorylation were higher in PF11 than in CK11. The combined results of carbon degradation, CAZymes, and glycolysis indicated that straw degradation in PF11 had reached the final stage. Drought alternation in rice fields can effectively promote straw decomposition. When the next rice crop was harvested and returned to the field, the straw returned from the previous cycle was already at the ultimate stage of decomposition.

The CH_4_ cycle is integral to the soil carbon cycle, and the processes of methanogenesis and CH_4_ oxidation processes directly affect greenhouse gas (GHG) emissions from paddy fields. Previous studies have shown that organic matter input can increase methane emissions in paddy fields, and that integrated rice-fish farming, through exogenous feed input, may potentially enhance methane emissions (Hou et al., 2025). However, in this work, we observed a contrasting pattern: the relative abundance of Euryarchaeota, the dominant methanogenic phylum, in PF11 was only 0.23-fold of that in CK11, indicating a substantial suppression of methanogenic communities and suggesting a reduction in methane emission potential under the straw return coupled rice-crayfish integrated system. This divergence from previous findings may be attributed to the combined effects of field drying and altered nitrogen availability, which collectively decreased methanogenic activity despite the organic matter inputs from straw and aquaculture sources.

The *mcrA* and *pmoA* genes, encoding methyl coenzyme M reductase and methane monooxygenase, respectively, drive the core metabolic processes of methanogenesis and methane oxidation and serve as key biomarkers for microbial-mediated methane metabolism. Their abundance ratio has been positively correlated with CH_4_ emissions (Kong et al., 2019). In this study, *mcrA* showed lower levels in PF than in CK; *pmoA* exhibited greater gene abundance in PF7 than in CK7, while PF11 showed slightly higher abundance than CK11. This pattern suggests that the integrated rice-crayfish system combined with straw return effectively decreased methanogenesis while enhancing methane oxidation, thereby reducing methane emissions potential. This finding is consistent with Jiang et al. (2024), who reported that rice-shrimp co-culture alleviated the increase in methane emissions caused by straw return. Notably, methanogens preferentially utilize acetate as a substrate during anaerobic fermentation, with 70%–80% of total methane production derived from acetate conversion (Pan et al., 2021); the suppression of methanogenic pathways in PF11 may therefore be partly associated with reduced acetate substrate availability.

Organic nitrogen degradation dominated N cycling, accounting for approximately 81.63%–84.14% of total N-cycling gene abundance across all treatments and indicating a high potential for converting organic nitrogen into NH_4_^–^-N for crop uptake (Silva et al., 2024). This pathway is closely coupled with organic carbon degradation and mainly involves the decomposition of aminobenzoate, nitrotoluene, branched-chain amino acids (valine, leucine and isoleucine) and lysine. This substrate profile indicated the active mineralization of both plant-derived lignin-like, and animal-derived, proteinaceous, organic nitrogen pools, reflecting the mixed organic matter inputs typical of the rice-crayfish system. Together with the enrichment of aromatic compound degradation in the C cycle, these results indicate that both carbon and nitrogen pools had entered the late stage of mineralization. Readily degradable substrates were largely depleted, and microbial communities shifted toward the utilization of more recalcitrant compounds, including aromatic nitrogenous compounds and branched-chain amino acids.

Straw return can enhance N_2_O emissions (Jiang et al., 2025), and aerobic-anaerobic alternation in paddy fields has also been reported to enhance gaseous N losses through nitrification-denitrification. On the one hand, the net N release under anaerobic conditions is generally assumed to be higher because anaerobes require less metabolic N under flooded conditions than under non-flooded or continuously aerated conditions. On the other hand, flooding may reduce fertilizer-derived and indigenous available N through enhanced immobilization (Akter et al., 2018), whereas organic fertilizer reduces these losses. Accordingly, the N fixation pathway was less pronounced in PF than in CK. In this work, nitrification converted NH_4_^+^ to NO_2_^–^ and NO_3_^–^ under aerobic conditions, with *nxrA/nxrB* as the key functional genes, and low gene abundances of *amoA* and *hao* indicated that ammonia oxidation to nitrite, the initial step of nitrification, was likely constrained. Nevertheless, the relatively high abundance of *nxrA/nxrB* suggested that the microbial potential for nitrite-to-nitrate oxidation remained intact. However, denitrification involved microbial reduction of nitrate (NO_3_^–^) and nitrite to NO, N_2_O, and N_2_, with *narG, nirS, norB, and nosZ* as key functional genes. Among these, *narG* encodes nitrate (NO_3_^–^) reductase, catalyzing the reduction of nitrate (NO_3_^–^) to nitrite (NO_2_^–^); *nirS* encodes nitrite reductase, catalyzing the reduction of nitrite to nitric oxide; and *norB* encodes nitric oxide reductase, reducing nitric oxide to nitrous oxide (N_2_O). Finally, *nosZ* encodes nitrous oxide reductase, which catalyzes the reduction of N_2_O to harmless dinitrogen (N_2_) (Sarkar et al., 2025; Silva et al., 2024; Wani et al., 2025). As shown in Figure 5F, In PF7, the abundance of *narG* was approximately 8.91 and 2.41 times that of *nirS* and *norB*, respectively. In PF11, this difference further increased, with *narG* abundance being approximately 24.04 and 4.22 times that of *nirS* and *norB*, respectively, indicating that nitrate (NO_3_^–^) reduction substantially outpaced subsequent nitrite reduction. Consequently, the nitrite accumulated from intensive nitrate (NO_3_^–^) reduction could be further oxidized to nitrate (NO_3_^–^) via *nxrA/nxrB*-dominated nitrification in paddy soil. In PF7, both nitrification and denitrification showed higher abundance than in CK7. By PF11, denitrification was lower than in PF7. This decrease was mainly related to field drying, which reduced SM content and increased oxygen availability, thereby suppressing denitrification activity.

### 4.4 Recommendations

(1) Soil total nitrogen content was lower than the national average, and nitrogen decreased substantially during the late rice-growing stage. Therefore, we recommend replacing conventional compound fertilizer with slow-release nitrogen fertilizer during the rice growing season to extend the nitrogen supply period, match the nutrient demand at different rice growth stages, reduce nitrogen leaching and volatilization losses, and ensure adequate nitrogen availability for rice growth.
(2) Two optional strategies are recommended for mitigating the environmental impacts of the ditch system: (a) introducing filter-feeding fish, e.g., silver carp and bighead carp (*Hypophthalmichthys molitrix*, *Hypophthalmichthys nobilis*), into the peripheral ditches to regulate water quality and increase economic output; or (b) adopting trenchless rice-crayfish farming systems in suitable areas to reduce the extent of prolonged flooding, lower methane emissions potential, and improve land use efficiency and field operability. This model is currently being promoted by the Chinese government (Wei et al., 2023).

### 4.5 Limitations

Several limitations should be acknowledged. Soil samples were obtained from one paddy field and one surrounding ditch; the five spatial subsamples within each plot were treated as pseudoreplicates, rather than true independent biological replicates. Hence, comparisons between PF and CK represent observational trends and cannot support formal statistical significance testing. Sampling was restricted to two time points (July and November, two key agronomic time-points), which limits our understanding of the continuous decomposition dynamics. Furthermore, the single-site nature of this study constrains the generalizability of our findings. Future research should incorporate rice-crayfish integrated systems without straw return and rice monoculture as control treatments, along with independent field replicates, to better clarify the decomposition processes of straw in integrated rice-aquaculture systems. In addition, given the limited treatment groups and strong collinearity among soil properties, the PLS-PM path coefficients, especially the boundary value of −1.00 for the TN to C-cycling genes path, should be considered exploratory rather than confirmatory.

## 5 Conclusion

This study evaluated how a rice-crayfish integrated system combined with straw return affected soil physicochemical properties, enzyme activities, and microbial functional genes associated with C and N cycling. The main conclusions are as follows.

(1) After the crayfish farming period, SOM and TN contents in the paddy field (PF7) were higher than in surrounding ditch (CK7), along with enhanced urease and cellulase activities, indicating that straw decomposition had entered an effective phase and that soil fertility had been substantially improved. By the end of the rice growing season (PF11), however, SOM content remained relatively stable, while TN content declined, indicating that nitrogen was continuously consumed by rice uptake, whereas organic matter accumulation was sustained through the gradual decomposition of straw residues and organic materials.
(2) Metagenomic analysis demonstrated that straw decomposition had progressed to the late stage by the end of the crayfish farming period (PF7), and had reached the final stage by the end of the rice growing season (PF11). Furthermore, the integrated system exhibited notable functional potential for mitigating greenhouse gas emissions.
(3) In the rice-crayfish integrated system with straw return constructed in this study, soil physicochemical properties and enzyme activities exhibited only weak associations with the functional gene profiles. The exception was TN, which exerted a strong negative direct effect on C-cycling genes, suggesting that N availability, rather than bulk fertility indices, constrains the functional potential of the community. This finding reveals the complexity of microbial community regulation mechanisms in the rice-crayfish integrated system and provides direction for future studies to identify the true ecological drivers of microbial community dynamics in this system.

## Data Availability

The data presented in the study are deposited in the NCBI SRA repository, accession number PRJNA1528484.

## Footnotes

1
https://github.com/voutcn/megahit

2
https://github.com/hyattpd/Prodigal

3
http://www.bioinformatics.org/cd-hit/

4
https://bowtie-bio.sourceforge.net/bowtie2/index.shtml

5
http://www.diamondsearch.org/index.php

6
http://www.diamondsearch.org/index.php

## References

[B1] Akter M. Deroo H. Kamal A. M. Kader M. A. Verhoeven E. Decock C. et al. (2018). Impact of irrigation management on paddy soil n supply and depth distribution of abiotic drivers. *Agricult. Ecosyst. Environ.* 261, 12–24. 10.1016/j.agee.2018.03.015

[B2] Gao X. Liu W. Li X. Zhang W. Bu S. Wang A. (2023). A novel fungal agent for straw returning to enhance straw decomposition and nutrients release. *Environ. Technol. Innov.* 30:103064. 10.1016/j.eti.2023.103064

[B3] Guo X. P. Yang Y. Niu Z. S. Lu D. P. Zhu C. H. Feng J. N. et al. (2019). Characteristics of microbial community indicate anthropogenic impact on the sediments along the Yangtze Estuary and its coastal area. China. *Sci. Total Environ.* 648, 306–314. 10.1016/j.scitotenv.2018.08.162 30121030

[B4] Han J. Song X. Fu H. Liu C. Yang F. (2024). Effects of the decomposition agent application on the physicochemical properties and microbial community structure of wheat straw-returning soil. *Environ. Technol. Innov.* 35:103668. 10.1016/j.eti.2024.103668

[B5] Han Y. Cui J. Guo P. Yang S. (2025). Remediation effects of straw combined with microbial agents on cinnamon soils with varying degradation based on metagenomics and untargeted metabolome. *Environ. Res.* 285:122649. 10.1016/j.envres.2025.122649 40846194

[B6] Hou Y. Jia R. Zhou L. Zhang L. Wei S. Li B. et al. (2025). Alterations in microbial-mediated methane, nitrogen, sulfur, and phosphorus cycling within paddy soil induced by integrated rice-fish farming. *J. Environ. Manag.* 388:126056. 10.1016/j.jenvman.2025.126056 40450938

[B7] Hua L. Gao Y. Li D. Yang Z. Liu Z. (2024). Effects of different straw return methods on farmland carbon footprint and water footprint. *Agriculture* 15:73. 10.3390/agriculture15010073

[B8] Jiang Y. Qin P. Li Z. Huang Z. Lin J. Shen X. et al. (2024). Effect of long-term straw returning on methane emissions in rice season of rice-crayfish coculture. *Chin. J. Eco-Agricult.* 32, 1451–1461. 10.12357/cjea.20240067

[B9] Jiang Z. Yang S. Pang Q. Abdalla M. Karbin S. Qi S. et al. (2025). Metagenomic insights into the influence of soil microbiome on greenhouse gas emissions from paddy fields under varying irrigation and fertilisation regimes. *J. Environ. Manage* 393:127129. 10.1016/j.jenvman.2025.127129 40896895

[B10] Jiao R. Dong R. Sun Q. (2015). *LY/T 1234–2015: Potassium Determination Methods of Forest Soils.* Beijing: National Forestry and Grassland Administration.

[B11] Jose V. L. More R. P. Appoothy T. Arun A. S. (2017). In depth analysis of rumen microbial and carbohydrate-active enzymes profile in Indian crossbred cattle. *Syst. Appl. Microbiol.* 40, 160–170. 10.1016/j.syapm.2017.02.003 28284522

[B12] Kong D. Li S. Jin Y. Wu S. Chen J. Hu T. et al. (2019). Linking methane emissions to methanogenic and methanotrophic communities under different fertilization strategies in rice paddies. *Geoderma* 347, 233–243. 10.1016/j.geoderma.2019.04.008

[B13] Li J. Lu J. Li X. Ren T. Cong R. Zhou L. (2014). Dynamics of potassium release and adsorption on rice straw residue. *PLoS One* 9:e90440. 10.1371/journal.pone.0090440 24587364 PMC3938734

[B14] Liu L. Yan Y. Zhang Q. Huang X. (2026). Analysis of the comprehensive utilization of rice straw and its potential in carbon sequestration and emission reduction based on bibliometrics. *Trans. Chin. Soc. Agricult. Eng.* 42, 281–290. 10.11975/j.issn.1002-6819.202509021

[B15] Liu Y. Li J. Cao Y. Lyu Q. Zhang G. Yan X. et al. (2026). Piled straw return: A novel strategy for mitigating greenhouse gas emissions in paddy fields. *Agricult. Ecosyst. Environ.* 399:110188. 10.1016/j.agee.2025.110188

[B16] National Aquatic Products Technology Extension Station, and Chinese Society of Fisheries (2025). Report on the development of China’s integrated rice-fish farming industry. *China Fish.* 8, 11–17.

[B17] Pan Y. Zheng X. Xiang Y. (2021). Structure-function elucidation of a microbial consortium in degrading rice straw and producing acetic and butyric acids via metagenome combining 16S rDNA sequencing. *Bioresour. Technol.* 340:125709. 10.1016/j.biortech.2021.125709 34375790

[B18] Peng X. Li R. Tang C. Teng W. Cheng K. Liu Z. et al. (2024). Effects of artificial humic acid on decomposition of returning straw and enhancement of carbon sequestration. *Appl. Soil Ecol.* 203:105619. 10.1016/j.apsoil.2024.105619

[B19] Qi D. Zhu J. (2026). The effects of the lengths of rice-crawfish rotations on the soil physicochemical properties of paddy fields. *J. Agricult. Food Res.* 25:102578. 10.1016/j.jafr.2025.102578

[B20] Ren Y. Xin J. Tian Y. Xu A. He L. Zhu L. et al. (2006). *Method for Determination of Soil Organic Matter, NYT1121.6-2006.* Beijing: The Ministry of Agriculture of the People’s Republic of China.

[B21] Rong F. Lin Y. Zhang M. Cai Z. Wu L. Chen F. (2025). Straw-derived bio-organic fertilizer enhanced ecosystem multifunctionality in acidic paddy by improving microbial community composition and functional diversity. *J. Environ. Manage* 390:126314. 10.1016/j.jenvman.2025.126314 40570423

[B22] Sarkar A. Patra R. K. Muthaiyan M. Mallik A. (2025). A metagenomic exploration of microbial distribution among the pond sediment, shrimp (*Penaeus monodon*) tissue and pond water: A special focus on nutrient cycling and shrimp viral pathogens. *J. Genet. Eng. Biotechnol.* 23:100568. 10.1016/j.jgeb.2025.100568 41386833 PMC12455100

[B23] Silva D. F. Cardoso E. J. Huang L. Erikson C. Silva A. M. Araujo V. L. et al. (2024). Functional genes related to n and p cycling in degraded and restored areas from brazilian drylands. *Appl. Soil Ecol.* 196:105316. 10.1016/j.apsoil.2024.105316

[B24] Tianjin Environmental Monitoring Center (2015). *Soil Quality-Determination of Total Nitrogen - Modified Kjeldahl Method, HJ 717-2014.* Tianjin: Tianjin Environmental Monitoring Center.

[B25] Wang M. Nan C. Wang Z. Chen F. (2007). *FDIS Determination of pH in Soil, NYT1377-2007*. Beijing: The Ministry of Agriculture of the People’s Republic of China.

[B26] Wani A. K. Qadir F. Elboughdiri N. Rahayu F. Saefudin Pranowo D. et al. (2025). Metagenomics and plant-microbe symbioses: Microbial community dynamics, functional roles in carbon sequestration, nitrogen transformation, sulfur and phosphorus mobilization for sustainable soil health. *Biotechnol. Adv.* 82:108580. 10.1016/j.biotechadv.2025.108580 40246210

[B27] Wei T. Wu M. Jiang J. Xi Y. (2023). Integrated rice-shrimp farming technology model without ring ditches. *China Fish.* 7, 60–62.

[B28] Wu L. Bai Y. Chen Y. Wei X. Wen N. Xu Z. et al. (2026). Straw return enhances soil multifunctionality by promoting protist-dominated microbial multitrophic interactions. *Soil Tillage Res.* 256:106903. 10.1016/j.still.2025.106903

[B29] Yan W. Xia M. Liu J. Han Z. Li Z. Rensing C. et al. (2026). Straw return improves soil multifunctionality by altering functional microbial diversity and abundance. *Agricult. Ecosyst. Environ.* 396:110015. 10.1016/j.agee.2025.110015

[B30] Yang G. Pan H. Lei H. Tong W. Shi L. Chen H. (2023). Dissolved organic matter evolution and straw decomposition rate characterization under different water and fertilizer conditions based on three-dimensional fluorescence spectrum and deep learning. *J. Environ. Manage* 344:118537. 10.1016/j.jenvman.2023.118537 37406492

[B31] Yang S. Chen L. Tan X. Pan X. Zeng Y. (2026). Dynamic change of soil organic carbon fractions improved by soil microorganisms at rice critical growth stages under long-term straw return in a double-cropped rice system in southern china. *Farm. Syst.* 4:100194. 10.1016/j.farsys.2025.100194

[B32] Zhang N. Bai L. Wei X. Li T. Tang Y. Zeng X. et al. (2025). Promoted decomposition in straw return to double-cropped rice fields controls soil acidity, increases soil fertility and improves rice yield. *Chem. Eng. J.* 509:161309. 10.1016/j.cej.2025.161309

[B33] Zhang Y. Li Y. Su F. Zhu C. Shan Y. (2026). The impact of freeze-thaw cycles on soil microbial biomass carbon and microbial community – a case study of northeastern brown soil in china. *J. Environ. Chem. Eng.* 14:121309. 10.1016/j.jece.2026.121309

[B34] Zhao D. Wu Y. Liu Z. Yu W. Xu Y. Wang Z. et al. (2026). Straw deep incorporation improves subsoil and rhizosphere soil microenvironment to increase wheat yield. *Eur. J. Agron.* 173:127921. 10.1016/j.eja.2025.127921

[B35] Zhao K. Xiao W. Wang N. Zhang X. Wang Y. Peng H. et al. (2025a). Carbon-nitrogen-phosphorus level regulation of potentially toxic elements and enzyme activities in paddy soil aggregates: Straw and organic fertilizer effects. *J. Environ. Chem. Eng.* 13:118166. 10.1016/j.jece.2025.118166

[B36] Zhao K. Zhao X. He L. Wang N. Bai M. Zhang X. et al. (2025b). Comprehensive assessment of straw returning with organic fertilizer on paddy ecosystems: A study based on greenhouse gas emissions, C/N sequestration, and risk health. *Environ. Res.* 266:120519. 10.1016/j.envres.2024.120519 39647690

[B37] Zhao Y. Chen L. Liu W. Yang H. Li S. Cui X. et al. (2026). Multi-source straw used as a resource can reduce environmental and economic burdens. *Environ. Impact Assess. Rev.* 118:108313. 10.1016/j.eiar.2025.108313

[B38] Zhou C. Li J. Song C. Ke W. Wang H. Yang S. et al. (2025). Microbial regulation of organic solid waste composting: Lignocellulose degradation (fertilization), process gas emissions, and containment of typical pollutants. *J. Environ. Manage* 393:127172. 10.1016/j.jenvman.2025.127172 40921105

[B39] Zhou G. Chang D. Gao S. Liang T. Liu R. Cao W. (2021). Co-incorporating leguminous green manure and rice straw drives the synergistic release of carbon and nitrogen, increases hydrolase activities, and changes the composition of main microbial groups. *Biol. Fertil. Soils* 57, 547–561. 10.1007/s00374-021-01547-3

