# Supplementary material for: Metagenomic insights into carbon and nitrogen cycling functions in an integrated rice-crayfish farming system with straw return
Source: Front Microbiol. 2026 Oct 26;17:1940285. doi: 10.3389/fmicb.2026.1940285 (PMC13745519; doi:10.3389/fmicb.2026.1940285)
Supplement: Supplementary file 1 [file Table_1.docx]

Table S1	The abundance of the top 20 microbial phylum levels

| Phylum | CK7 | PF7 | CK11 | PF11 | PF7/CK7 | PF11/CK11 | PF11/PF7 |
| --- | --- | --- | --- | --- | --- | --- | --- |
| Pseudomonadota | 1145825 ± 166436 | 1657400 ± 292317 | 1473863 ± 87437 | 1543536 ± 161124 | 1.45 | 1.05 | 0.93 |
| unclassified Bacteria phylum | 770236 ± 35745 | 769135 ± 114511 | 776471 ± 57309 | 964961 ± 52697 | 1.00 | 1.24 | 1.25 |
| Thermodesulfobacteriota | 717859 ± 124200 | 373744 ± 92541 | 750600 ± 79906 | 265730 ± 66182 | 0.52 | 0.35 | 0.71 |
| Acidobacteriota | 409913 ± 69436 | 514074 ± 162032 | 418037 ± 62772 | 664080 ± 73667 | 1.25 | 1.59 | 1.29 |
| Bacteroidota | 719494 ± 130927 | 248615 ± 87297 | 300319 ± 57939 | 68370 ± 23140 | 0.35 | 0.23 | 0.28 |
| Chloroflexota | 241725 ± 17350 | 275108 ± 17779 | 399110 ± 39679 | 370893 ± 60485 | 1.14 | 0.93 | 1.35 |
| Myxococcota | 199816 ± 34256 | 175096 ± 49024 | 226435 ± 47179 | 124011 ± 33989 | 0.88 | 0.55 | 0.71 |
| Nitrospirota | 104833 ± 42211 | 185860 ± 54438 | 110973 ± 46364 | 278197 ± 48914 | 1.77 | 2.51 | 1.50 |
| Verrucomicrobiota | 217039 ± 36363 | 162443 ± 10886 | 137293 ± 23335 | 163419 ± 19242 | 0.75 | 1.19 | 1.01 |
| Actinomycetota | 88653 ± 4055 | 114139 ± 26273 | 77398 ± 14565 | 128648 ± 25798 | 1.29 | 1.66 | 1.13 |
| Gemmatimonadota | 66903±12962 | 183161±44526 | 41170±2122 | 183738±15707 | 2.74 | 4.46 | 1.00 |
| Euryarchaeota | 74576±15291 | 59934 ±8363 | 173112±18525 | 39043±12,503 | 0.80 | 0.23 | 0.65 |
| Cyanobacteriota | 58,574 ±9,870 | 138,914 ±17,285 | 25,915 ±4,245 | 7,894 ±341 | 2.74 | 0.30 | 0.057 |
| Candidatus Rokubacteria | 39617 ± 22510 | 39390 ± 24926 | 84801 ± 75469 | 74842 ± 35038 | 0.99 | 0.88 | 1.90 |
| Planctomycetota | 63973 ± 15375 | 47579 ± 5523 | 70333 ± 15259 | 35098 ± 3164 | 0.74 | 0.50 | 0.74 |
| Ignavibacteriota | 64974 ± 14286 | 76837 ± 23445 | 30421 ± 9040 | 36350 ± 12028 | 1.18 | 1.19 | 0.47 |
| Candidatus Aminicenantes | 31542±1337 | 62836±16248 | 77705±18718 | 65623±10855 | 1.99 | 0.85 | 1.04 |
| candidate division NC10 | 21159 ± 6504 | 27410 ± 7780 | 63054 ± 37564 | 42245 ± 6694 | 1.30 | 0.67 | 1.54 |
| Nitrososphaerota | 6999 ± 3868 | 53204 ± 40173 | 6019 ± 6135 | 63648 ± 12872 | 7.60 | 10.57 | 1.20 |
| Uroviricota | 27580±8355 | 19843±5056 | 58161±9456 | 8029±1227 | 0.72 | 0.14 | 0.40 |
